# Increased Levels of (p)ppGpp Correlate with Virulence and Biofilm Formation, but Not with Growth, in Strains of Uropathogenic *Escherichia coli*

**DOI:** 10.3390/ijms24043315

**Published:** 2023-02-07

**Authors:** Monika Karczewska, Patryk Strzelecki, Krystyna Bogucka, Katarzyna Potrykus, Agnieszka Szalewska-Pałasz, Dariusz Nowicki

**Affiliations:** 1 Department of Bacterial Molecular Genetics, Faculty of Biology, University of Gdansk, Wita Stwosza 59, 80-308 Gdansk, Poland; 2 Institut de Physique et Chimie des Matériaux de Strasbourg, Université de Strasbourg, CNRS, UMR7504, 23 rue du Loess, CEDEX 2, F67034 Strasbourg, France

**Keywords:** UPEC, ppGpp, stringent response, virulence, UTI, biofilm, *E. coli*, *Galleria mellonella*, phenotype, pathogenicity, infection, antibiotic resistance

## Abstract

Urinary tract infections are one of the most frequent bacterial diseases worldwide. UPECs are the most prominent group of bacterial strains among pathogens responsible for prompting such infections. As a group, these extra-intestinal infection-causing bacteria have developed specific features that allow them to sustain and develop in their inhabited niche of the urinary tract. In this study, we examined 118 UPEC isolates to determine their genetic background and antibiotic resistance. Moreover, we investigated correlations of these characteristics with the ability to form biofilm and to induce a general stress response. We showed that this strain collection expressed unique UPEC attributes, with the highest representation of FimH, SitA, Aer, and Sfa factors (100%, 92.5%, 75%, and 70%, respectively). According to CRA (Congo red agar) analysis, the strains particularly predisposed to biofilm formation represented 32.5% of the isolates. Those biofilm forming strains presented a significant ability to accumulate multi-resistance traits. Most notably, these strains presented a puzzling metabolic phenotype—they showed elevated basal levels of (p)ppGpp in the planktonic phase and simultaneously exhibited a shorter generation time when compared to non-biofilm-forming strains. Moreover, our virulence analysis showed these phenotypes to be crucial for the development of severe infections in the *Galleria mellonella* model.

## 1. Introduction

Urinary tract infections (UTIs) are among the most frequently occurring major bacterial infections worldwide, and their significant increase in incidence in recent years is alarming [1,2,3]. It is estimated that 150 million UTIs occur annually worldwide, with significant morbidity and high treatment costs affecting the efficiency of national health care systems [4]. In the US alone, each year, more than 10 million doctor office visits, 2 million emergency department visits, and 100,000 hospitalizations are associated with UTIs [5,6]. Those at high risk are newborns, pre-school girls, sexually active women, and older people of both sexes [7]. UTIs mainly affect the female population, which is related to the structure of the urinary tract. A cohort study among 2000 women representing the US population corroborates this finding [8,9].

The prevalence of UTIs results in rampant use of antibiotics, which impacts the spread of resistance. This is a clinical problem, especially in recurrent UTIs [10]. Therefore, empirical treatment of emergency room patients is a challenge for clinical urology and requires the constant evaluation of bacterial susceptibility to antibiotics

UTIs have been associated with such bacterial species as Pseudomonas aeruginosa, Acinetobacter baumannii, Staphylococcus aureus, and those belonging to the Enterobacteriaceae family, such as Klebsiella pneumoniae, Proteus mirablis, Citrobacter, and Enterobacter, as well as some Candida species [11,12]. However, uropathogenic Escherichia coli (UPEC) are most commonly responsible for about 80% of uncomplicated UTIs, 95% of out-of-hospital infections, and 50% of hospital-acquired infections [13]. UPEC are included in the ExPEC pathotype, along with other E. coli isolates involved in various extraintestinal infections. UPEC strains have evolutionarily adapted to colonize a niche in the urinary tract. They harbor genetic virulence factors (VF) on pathogenicity-associated islands (PAIs) [14], contributing to bacterial pathogenesis and distinguishing them from commensal strains [15]. Phylogenetic analyses have shown that E. coli strains classify into four major phylogenetic groups (A, B1, B2, and D), of which those belonging to groups B2 or D are most often responsible for extraintestinal infections [16]. Eight phylogenetic groups are currently recognized by the classification established by Clermont: seven (A, B1, B2, C, D, E, F) belong to E. coli sensu stricte, while a distinct example is Escherichia cryptic clade I [17].

UPEC virulence requires coordinated expression of multiple genes that facilitate adhesion and colonization in the urinary tract. One can distinguish between VFs bound to the bacterial cell surface and those that are secreted and exported to the site of action. Adhesion to tissues within the urinary tract is the most important step in pathogenicity and is enabled by adhesion factors. These include various types of fimbriae (types 1, P, S, F1C), fimbrial Dr adhesins, as well as afimbrial Afa adhesins and PapC. Among the toxins that help bacteria spread to deeper tissues after disrupting cell integrity and that can cause an inflammatory response, the most important are the lipoproteins—α-hemolysin (HlyA) and cytotoxic necrosis factor 1 (CNF1) [18,19]. Moreover, the main component of the cell wall—lipopolysaccharide (LPS), antigen O and K—is an endotoxin that helps suppress the host immune system [20]. The availability of iron is very limited in the urinary tract, so in order to survive in this environment, bacteria encode proteins of iron-acquiring systems. UPEC strains are particularly well-equipped with the factors required for biosynthesis and uptake of the following siderophores: enterobactin, aerobactin, yersiniabactin and salmochelin, which they then transport into their cytoplasm through the Sit system (siderophore-iron transporter proteins, SitABCD) [21]. 

Biofilms are increasingly recognized as major contributors to pathogenicity, recurrence, chronicity, and recalcitrance-to-treatment of UTIs, as well as many other diseases. Biofilm is defined as a structured formation of bacterial cells adhering to a surface, surrounded by a polymeric matrix that they have produced [22]. Biofilm formation can be considered to be another virulence factor for UPEC strains, which promotes their survival in the urinary tract by protecting against the cleansing effects of hydrodynamic forces, host defense mechanisms, and antibiotics [23]. Currently, the ability to form a biofilm is not well understood, and several factors are generally considered to be involved in its formation, including adhesins and specific proteins such as antigen 43 [24]. Thus, the problem of frequent UTI recurrences and the tendency for UTIs to develop into a chronic form has been associated with biofilm formation during infection [25].

There is a general consensus in the research that the so-called stringent response is involved in the control of the pathogenicity of various bacterial species as a mechanism of bacterial adaptation towards changing environmental conditions through the modulation of gene expression. It is involved in processes related to growth, stress, starvation, and survival, and for many bacterial species, the stringent response is required for efficient expression of the virulence factors [26,27,28]. In the broadest terms, the stringent response is the global response of a cell to stress conditions resulting in inhibition of most processes requiring energy consumption [26]. The key molecules in the stringent response are the small-nucleotide alarmones, ppGpp, and pppGpp (guanosine tetra- and pentaphosphates) collectively referred to as (p)ppGpp. These alarmones are synthesized in *E. coli* by two enzymes. One is RelA, which responds to amino acid starvation, and the other is the SpoT enzyme, which is responsible for the synthesis of (p)ppGpp during other types of stresses and deprivation, but additionally is involved in the hydrolysis of (p)ppGpp [26,29]. However, the importance of (p)ppGpp in the exponential growth rate control has also been presented [30]. The (p)ppGpp signaling controls global metabolic changes in response to environmental fluctuations and regulates such processes as biofilm formation, quorum sensing, adaptive processes, or bacterial virulence [31]. The entrance of cells into the stationary phase of growth is correlated with increased accumulation of intracellular (p)ppGpp [26]. This is caused by environmental constraints on growth, such as the depletion of nutrients or excess intermediate metabolites secreted into the environment, and it activates the effector proteins RelA and SpoT to synthesize the alarmones. This leads to changes in the expression of a number of metabolism-related genes, as well as the activation of the regulator FimB, which controls the phase variation of type 1 fimbriae in *E. coli* [32,33]. Type I fimbriae mediate the adhesion and invasion of UPEC and also are an important factor at the initial stages of biofilm formation [9,34,35]. 

Due to the prevalence of UTIs and their potential for recurrence and subsequent medical complications, UPEC infections are a serious health problem worldwide. The main aim of this study was to examinate the genetic diversity of UPEC isolates from UTI patients from northern Poland. Furthermore, we conducted a phylogenetic clustering based on Clermont’s classification, as well as testing evaluating susceptibility to basic antibiotics. In our thorough approach, we also investigated the UPEC strains’ biofilm-forming ability (assessed by differentiation on Congo red agar), and evaluated their metabolic phenotypes by measuring the stringent response alarmones’ basal levels, as well as assessing the growth rates of selected strains. Then, strains showing specific phenotypes were subjected to virulence analysis in a surrogate infection model of *Galleria mellonella*.

## 2. Results

### 2.1. Distribution of Virulence Factors and Phylogenetic Clustering of Isolates

**Figure 1:**
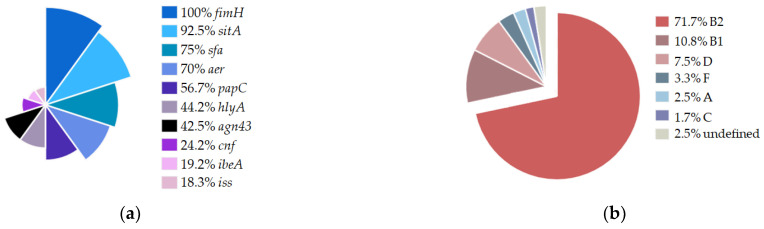
Genetic characteristics of the UPEC collection. (**a**) Percentage distribution of virulence factors identified by PCR in UPEC isolates. (**b**) Percentage distribution of phylogroups determined by phylogenetic typing using the quadruplex PCR method [17].

First, we decided to evaluate the genetic background of the tested isolates, employing PCR analysis with previously published primer pairs regarding the above described virulence factors (VFs). According to the results of our genetic analysis, a relatively high distribution of VFs among the isolates studied (Figure 1a) is an important phenotypic characteristic, as one or more VFs were expressed in 100% of the strains. All of them encoded the *fimH* gene, and among the other factors analyzed, the most frequently identified were *sitA* (92.5%), *aer* (75%), and sfa (70%). The ability to express HlyA and/or PapC proteins, as well as antigen 43 occurred at a medium level (42.5–56.7%). In contrast, the lowest distribution was obtained for the *cnf*, *ibeA*, and *iss* genes (24.2%, 19.2%, and 18.3%, respectively). For 117 of the 120 strains, phylogroups were determined by extended quadruplex PCR. Of the 8 phylogroups described [17], 6 were identified in our collection (Figure 1b). A large number of isolates was assigned to group B2 (71.7%), followed by groups B1 (10.8%) and D (7.5%). UPEC isolates from groups F, A, and C were less frequently represented (3.3%, 2.5%, and 1.7%, respectively).

**Figure 2:**
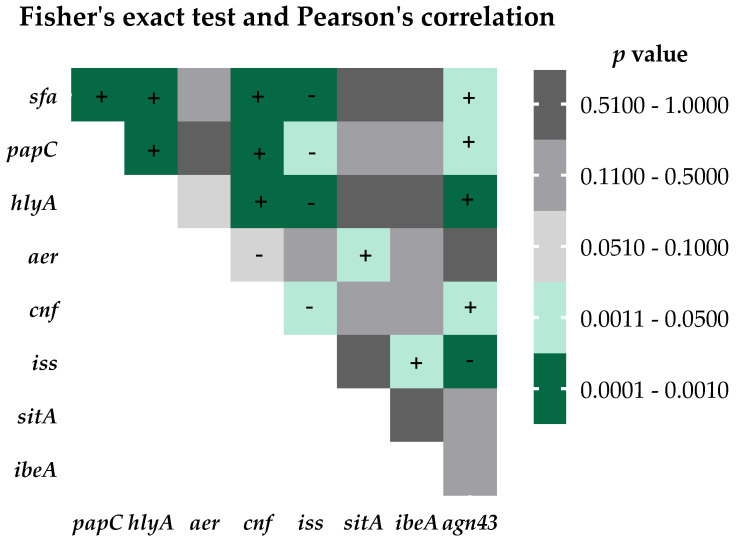
Co-occurrence matrix of virulence factors. Statistically significant results (Fisher’s exact test, *p* < 0.05) are shown in green, while non-significant correlations are shown in gray. The direction of correlations was determined using Pearson’s test, where “+” indicates a positive correlation and “-” a negative correlation (*p* < 0.05). The matrix presents horizontal values vs. vertical values.

Based on the findings above, a series of analyses were carried out to determine the co-occurrence of virulence factors in relation to each other. Correlations were statistically confirmed with Fisher’s exact test (Figure 2). In particular, many correlations were determined for the *sfa*, *papC*, and *agn*43 genes. The least correlated with other VFs were the *ibeA*, *sitA*, and *aer* genes (Figure 2). For each pair, the direction of correlation was determined to be either positive or negative, using Pearson’s test (Figure 2). A strong correlation was observed for the presence of the *cnf* gene, which was always present with the *hlyA* gene, while the presence of *hlyA* was not dependent on the presence of *cnf* (See supplementary material for details). 

Moreover, by including Clermont’s classification in the analysis, we observed that different phylogenetic groups had different quantitative distributions of VF. The B2 group had the highest mean value (6.01), the A, C, and D groups had intermediate values, while B1 had the lowest value. Thus, the correlation between individual virulence factors and Clermont’s phylogroups was analyzed. Some peculiarities should be noted, such as the occurrence of the *cnf* gene exclusively in the B2 phylogroup. Similarly, the *hlyA* or *ibeA* gene sequences are present only in this group, except for two isolates (Table 1). Due to the size of the groups, only the differences between the largest B1 and B2 groups were evaluated. Statistical analysis shows that these groups differ in the distribution of all analyzed genes except for *fimH*, *ibeA*, and *aer*, with the *fimH* sequence present in all strains, regardless of the group (Table 1).

**Figure 3:**
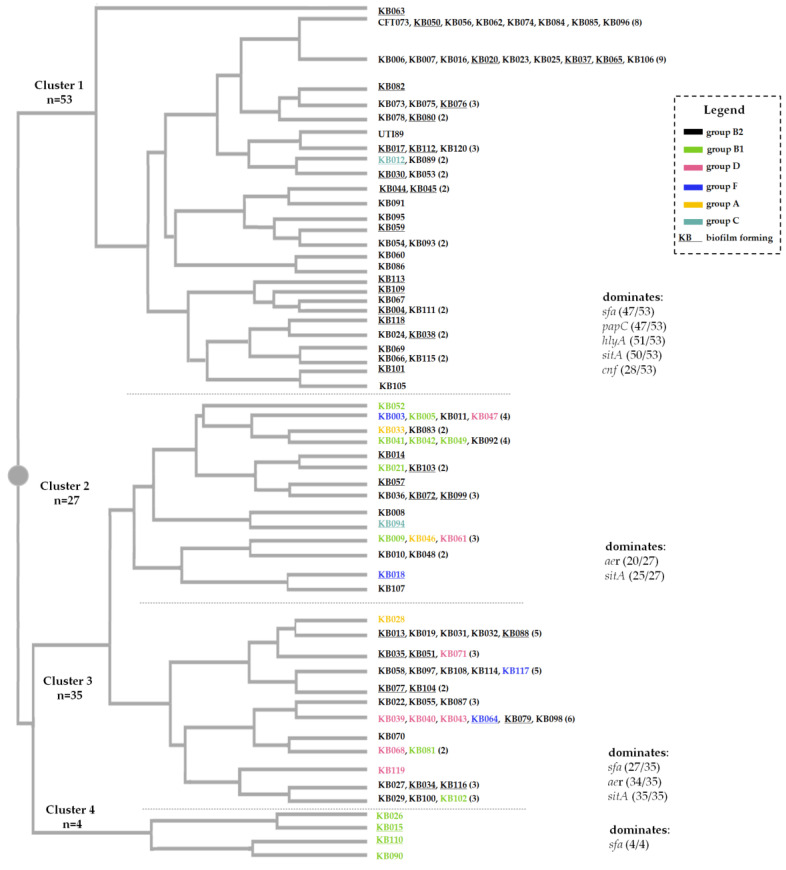
Genetic relationships among UPEC strains. The dendrogram was created by the UPGMA method using the DendroUPGAMA server [36]. Colors mark the different phylogenetic groups identified, and strains that form biofilm on Congo red agar are underlined. The dashed lines divide the dendrogram into four clusters. The first cluster brings together all strains encoding the *cnf* gene.

The high genetic diversity of the strains is shown on a dendrogram (Figure 3). Based on the UPGAMA method, a dendrogram was constructed in which 120 strains were grouped, based on the presence of virulence genes. Altogether, 51 different virulotypes were delineated. The most common genotypes were: (i) *fim, sfa, papC, hlyA, aer, sitA,* and *agn43;* (ii) *fim, sfa, papC, hlyA, aer, cnf, sitA,* and *agn43;* (iii) *fim, sfa, papC, aer,* and *sitA;* (iv) *fim, sfa, papC, cnf, sitA,* and *agn43;* and (v) *fim, sfa, cnf, sitA,* and *agn43,* and these accounted for 27.5% of the population studied. It is also worth noting that the most common virotypes present are characterized by a large number of virulence factors. Overall, the occurrence of three or more VFs was recorded in 115 isolates, which represents 95.8% of the studied population. 

### 2.2. The Biofilm-Forming Phenotype Does Not Correlate with Genetic Background

Since the ability to form biofilm is an important pathogenicity factor, we decided to evaluate our collection according to this characteristic. In our collection, biofilm production on Congo red agar (CRA) was reported in 32.5% (n = 39) of UPEC. Intensely black colonies were present among 9 strains, 11 were moderately positive, and 19 were weakly positive (collectively referred to as CRA+). CRA+/− strains and phylogenetic groups are marked on the dendrogram (Figure 3). The link between biofilm formation and Clermont’s phylogenetic classification was assessed. The phenotype of colonies on CRA (CRA+, CRA−) did not correlate with the phylogenetic groups, which was confirmed using Fisher’s exact test (Table 2). In addition, whether this trait could be correlated with any of the virulence genes was also assessed. The analysis showed a significant co-occurrence of the IbeA factor in biofilm forming strains (*p* = 0.0025). No correlation was observed for other VFs.

### 2.3. Biofilm Forming Abilities Correlate with a Multi-Resistant Phenotype

Since bacteria forming biofilms usually display multidrug resistance, we decided to assessed this ability using our collection. In this study, we analyzed the susceptibility of UPEC strains to 16 antibiotics (Table S1). Resistance was noted in 53 strains (44.2% of the population), and among them almost half (52.8%) showed resistance to more than one antibiotic. Most strains displayed lack of sensitivity to ampicillin (92.5%). Chemotherapeutics (norfloxacin, ciprofloxacin, nitrofurantoin, trimethoprim/sulfamethoxazole) also had a high resistance profile (58.5%). Of all the antibiotics evaluated, cefoxitin, meropenem, ceftibuten and fosfomycin were the most effective agents against the strains from our collection. 

Whether the incidence of resistance was related to the age of the patient from whom the strain was isolated was also assessed. According to the phenotypic resistance evaluation, it was noted that the resistant strains (56.7%) were derived from individuals who were 50 years of age and older on the day of bacterial isolation. Moreover, these strains represented 71.7% of all resistant isolates in the collection. For the second group (below 50 years old), these percentages were 31.3% and 28.3%, respectively (*p* = 0.0082). 

The relevance of the consequences of biofilm formation and drug resistance to the severity of the infection [37] prompted us to delve deeper into the characteristics of the strains and compare the different traits among them. We showed that the multidrug resistance profile of isolates was significantly more frequent in the group of strains capable of biofilm formation on CRA when compared to the non-biofilm forming group (Table 3).

### 2.4. Higher Basal (p)ppGpp Level Results in Shorter Doubling Time in UPECs 

**Figure 4:**
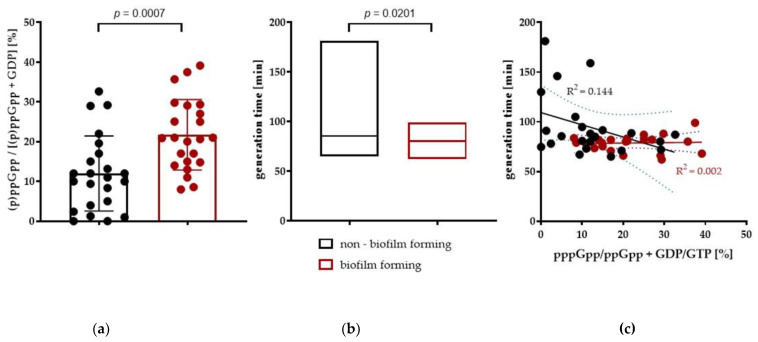
Phenotypic evaluation of the UPEC strains. Non-biofilm forming (n = 24) and biofilm-forming (n = 23) strains were grown aerobically in MOPS minimal medium (0.2% glucose), with shaking. (**a**) The relative accumulation of the (p)ppGpp alarmones was assessed by the ^32^P labeling of nucleotides and revealed chromatographically on PEI cellulose plates by TLC in 1.5M KH_2_PO_4_, pH 3.4. The presented data were collected at an exponential phase of growth (A600 = 0.3) (**b**) Doubling time was determined spectrophotometrically with a plate reader. (**c**) Linear regression plot obtained by combining both parameters (generation time and alarmone accumulation), with R2 values indicated. Results are obtained from at least three independent experiments and are expressed as the mean with SEM. Statistical significance was determined by unpaired Student’s t-test.

It has been shown that (p)ppGpp levels negatively correlate with growth rate [30]. We wondered whether this would be a case here as well. Analysis of the basal level of the stringent response alarmone accumulation, (p)ppGpp, in the studied *E. coli* strains showed significant differences between the individuals strains. Some of them presented higher intrinsic levels of (p)ppGpp, and interestingly, this occurred under growth conditions without nutrient limitation. This trait turns out to be correlated with their ability to form biofilm on CRA, indicating a direct or indirect effect of (p)ppGpp on biofilm formation (Figure 4b). Another characteristic that differentiates groups of CRA+/− strains is the generation time (Figure 4b), with the CRA+ strains showing faster growth rates than the CRA- strains. The correlation of these two characteristics with the determined regression line is shown in Figure 4c.

### 2.5. Biofilm-Forming UPECs Are More Virulent to Galleria Mellonella Larvae

**Figure 5:**
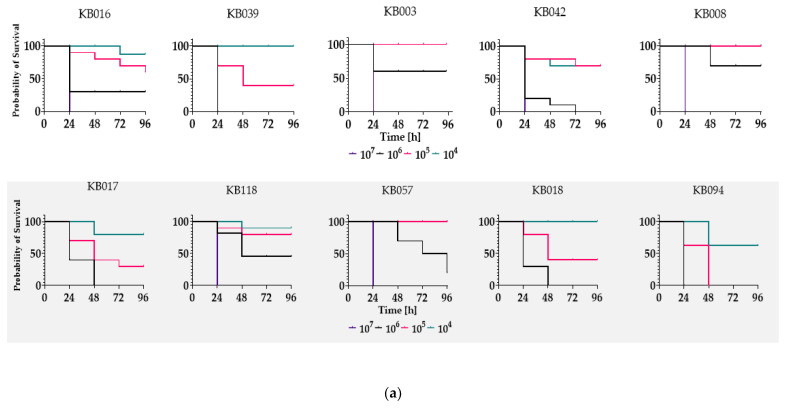

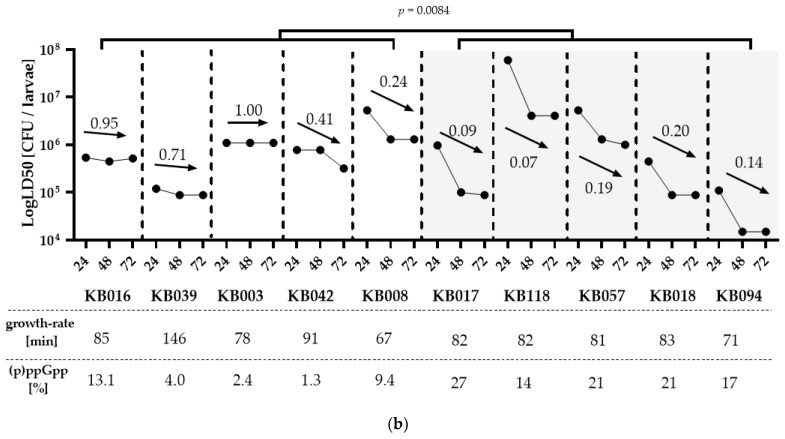
Evaluation of virulence of CRA+/− UPEC strains. Evaluation of virulence of biofilm-forming (gray part of panel) and non-biofilm-forming (colorless part of panel) UPEC strains in a surrogate infection model of *G. mellonella*. The 5 biofilm producers chosen were selected based on the greatest ability to form biofilm, and the 5 non-biofilm forming samples were randomly selected. (**a**) Kaplan–Meier survival curves of *G. mellonella* after injection of 10^4^–10^7^ cells/larva. (**b**) LD50 values calculated between 24 and 72 h. Percentage changes in LD50 between the 1st and 3rd days are indicated by arrows, with their value above them. The values of growth rate (min) and alarmone relative concentration (%) in particular strains are indicated under the plot.

Finally, in order to determine if the biofilm-forming UPEC are more virulence than non-forming examples we employed a *G. mellonella* larvae model. As discussed in our previous work [38] this larvae serves as a surrogate model of infection. The model is cost efficient, effective, reliable, and does not raise ethical concerns. Moreover, its use, has been described to study UPEC virulence [39,40]. The larvae were injected with a bacterial inoculum (10^4^ to 10^7^ cfu/larvae) to determine the mortality rate of insects exposed to each isolate. Five strains from each of the CRA+/− groups were tested. The survival of larvae was monitored for several days, and the results were collected in the form of Kaplan–Meier curves (Figure 5a). The *G. mellonella* larvae showed different survival profiles, depending on the strain used. However, in all cases, only the relatively high infectious dose 10^7^/larvae resulted in 100% mortality rate within 48 h. In addition, a quantified LD50 value between 24 and 72 h post-inoculation and the LD50 reduction level (LD50 72h/LD50 24 h) were calculated for each isolate (Figure 5b). The virulence of biofilm-forming isolates was higher in this model than that in the non-biofilm-forming samples.

## 3. Discussion

The goal of this work was to comprehensively characterize clinical isolates of the UPEC strains. Our studies show that these strains present characteristic features of their genus, but we also demonstrated a new characteristic phenotypic pattern for the studied collection, which should be further explored.

Among the 9 genes coding for the virulence factors tested, the most frequently detected were *fimH* (100%), *sitA* (92.5%), *aer* (75%), and *sfa* (70%). Such a high distribution of the *fimH* gene was also confirmed in studies conducted in Ethiopia, Romania, Mongolia, Iran, Mexico, and China [13,41,42,43,44,45,46], which may indicate a key role of this factor in the development of UTI. The percentage of other VFs (Sfa, PapC, HlyA, CNF1) that characterize the strains in the studied collection is on par with another study conducted in Poland [47], while in other countries, the share of these factors varies [13,48,49,50,51,52]. A high percentage of sequences encoding SitA and Aer characterized the UPEC studied, and this observation is in line with the results obtained in other studies [53,54,55,56,57]. The presence of such proteins indicates that, despite the environment in the urinary tract where iron access is limited, UPEC strains are able to survive and develop UTI. Additionally, the presence of the two genes in our study was significantly correlated with each other (Figure 2). Moreover, we observed a correlation between the genes of toxins related with a pathogenicity island, namely *hlyA* and *cnf*. The *cnf* gene was recently shown [58] to be dependent on *hlyA* presence, while *hlyA* occurrence is independent of *cnf*. The invasive protein IbeA and the serum survival enhancing factor Iss were the least frequent, with similar percentages described by Derachshandeh et al. [59]. The described virulence factors are crucial for triggering urinary tract infections; however, UPEC strains can differ significantly in terms of genetic background. This was confirmed by the fact that in the studied population of 120 strains, as many as 51 different virulotypes were obtained.

Pathotypes of virulent *E. coli* strains are often associated with a particular phylogroup. We determined that the predominant group in the collection was group B2 (71.7%), followed by groups B1 (10.8%) and D (7.5%). It is noteworthy that virulent extraintestinal strains mainly belong to groups B2 and D [60]. Similar findings were obtained in other studies, in which the B2 group was predominant [45,49,61,62,63]. Different phylogenetic groups expressed different average VF counts. Group B2 had the highest score of 6.01, groups A, C, and D were intermediate, while B1 had the lowest number of VF genes. A similar relationship for groups B2 and B1 was reported recently by Rezatofighi et al. [64]. A positive correlation of VFs and those phylogroups was further observed in studies from the early 2000s [65,66].

The increasing prevalence of UTIs results in the overuse of broad-spectrum antibiotics, such as fluoroquinolones, cephalosporins, and aminoglycosides, which contributes to increased antibiotic resistance [10]. Resistant strains, including those not susceptible to more than one antibiotic, were also present in the population of isolates studied. The highest resistance was attributed to ampicillin (92.5%) and chemotherapy (norfloxacin, ciprofloxacin, nitrofurantoin, trimethoprim/sulfamethoxazole; 58.5%). In their study, Oliveira et al. reported 59% of strains resistant to at least one of the antimicrobial agents tested, including the highest for ampicillin (51%) and trimethoprim-sulfamethoxazole (44%) [50]. Likewise, in an Australian study on antimicrobial use and bacterial resistance conducted in 2014–2015, the highest resistance rate for *E. coli* was also recorded for ampicillin (53.2%), followed by trimethoprim (31.3%). It is also worth mentioning that the percentage of resistant isolates was higher in 2015 when compared to 2014 [67]. A high rate of ampicillin-resistant strains was also obtained in other studies [47,52,68]. Currently, many strains resistant to multiple antibiotics remain susceptible to fosfomycin (FOS), even in geographic regions where its use is widespread. This antibiotic has shown efficacy, both in vitro and in vivo, in treating UTIs caused by ESBL-producing *Enterobacteriaceae* and vancomycin-resistant enterococci [69]. In this study we showed that none of the isolates, including the multidrug-resistant ones, were resistant to FOS (as well as to ceftibuten, meropenem, and cefoxitin). Similar results with low rates of resistance or complete sensitivity to FOS have been obtained in other studies at the Charlotte Maxeke Johannesburg Academic Hospital, among others [70,71,72]. These findings suggest that fosfomycin is still an effective treatment for urinary tract infections, but should be used with caution, and studies are needed to monitor the resistance status of strains isolated from patients with urinary tract infections.

It has been previously described that bacterial biofilm protects bacteria from antibiotics and is involved in many diseases [73]. Biofilms are communities of bacteria encased in a polymeric matrix that protects resident bacteria from both the antibiotics and the host immune effectors, among many other stressors and potential means of eradication. Our results confirm the correlation between biofilm-forming ability and antibiotic resistance phenotype; however this tested was conducted under non-biofilm-forming conditions. Although the percentage of resistant strains in the CRA+/− groups occurred at a similar level, biofilm-forming isolates were more likely to show resistance to more than one antibiotic. Bacteria residing within a biofilm are canonically highly resistant to antibiotic-mediated killing and are overall antimicrobial resistant, with resistance often exhibited toward multiple classes of antibiotics. Such a result was obtained in a study by Katongole et al. [74]. The authors also found no correlation of biofilm production with any of the virulence factors studied. Our study coincides with these observations, with the exception of the *ibeA* gene (this sequence was not studied in the Katangole et al. experiment). Furthermore, in another study conducted in Poland, no relationship between the rate of biofilm formation and the presence of adhesion factors or toxin genes was determined [47]. Only the aerobactin gene was significantly associated with strong biofilm production. However, it is worth mentioning that in this study, the assessment of biofilm formation capacity was investigated with a test other than CRA.

Biofilm is formed by the aggregation of bacterial cells, surrounded by a matrix composed of eDNA, proteins, and exopolysaccharides. These polysaccharides facilitate adhesion to the cell surface and protection from environmental stress. This is accompanied by a high concentration of 3′, 5′-cyclic diguanosine monophosphate [c-di-GMP] [75]. Degradation of c-di-GMP occurs due to cyclic phosphodiesterase (PDE) activity of the Yybt protein [76]. Interestingly, Rao et al. found that the bacterial regulator ppGpp is a potent inhibitor of the DHH/DHHA1 domain found in YybT, suggesting that YybT is under tight control during the stringent response [77]. In 2017, Jones et al. described that if c-di-GMP regulates the production of biofilm matrix polysaccharides, Congo red staining can be used as an indirect measurement of elevated c-di-GMP production in bacteria [78]. Hence, if elevated levels of (p)ppGpp contributes to increased levels of c-di-GMP, we concluded that strains with black colonies on CRA may produce increased levels of the alarmones. Our study confirmed the assumption that CRA+ strains have significantly higher basal levels of alarmones (Figure 4).

Moreover, in addition to the effect of (p)ppGpp on biofilm formation, alarmone production has also been linked to the expression of virulence factors or antibiotic tolerance. There are reports suggesting that UPEC strains with high intrinsic levels of (p)ppGpp exhibit higher levels of pathogenicity against the host [79,80]. To test whether reports of increased virulence of such strains would also be confirmed in our study, we screened the infectivity of UPEC differing in (p)ppGpp levels in a *G. mellonella* model. The results confirmed the initial assumption of increased virulence of CRA+ strains exhibiting higher intrinsic levels of (p)ppGpp (Figure 5). In laboratory *E. coli* strains, the cellular level of (p)ppGpp is closely related to their growth rate. The increase in the (p)ppGpp level is observed in response to stress factors, such as starvation, and results in a rapid accumulation of high alarmones concentrations (600–1000 pmol OD^−1^) [81]. This leads to the inhibition of rRNA, DNA, and protein synthesis and in turn, yields slower growth rates. However, to date, there are still gaps in the knowledge regarding the role of the basal level of (p)ppGpp, which occurs in the cell under optimal growth conditions, and how it can affect cell physiology.

The high heterogeneity of the tested strains, demonstrated by genetic-phenotypic analysis, may arise from the differences in (p)ppGpp levels between clinical isolates. These differences could be due to either the polymorphism of molecular targets of (p)ppGpp or proteins interacting with RelA and SpoT, including regulatory proteins or the polymorphism of RelA and SpoT proteins themselves. Stringent response exhibits pleiotropic effects, triggering vast metabolic adjustments in bacteria to adapt to a challenging environment. However, alarmones are also involved in VFs expression; therefore, we hypothesize that observe variations in (p)ppGpp levels among particular strains served as an adaptation mechanism, increasing competitiveness; however, this theory still awaits verification. Moreover, the observed common assumptions do not always correlate with the expected phenotype. As an example, enterohemorrhagic *E. coli* (EHEC) strains have a positive correlation between (p)ppGpp and the transcription of virulence factors encoded on the LEE pathogenicity island, while an inverse correlation is observed between high levels of (p)ppGpp and the Shiga toxin production [82]. Similarly, it is well known that high levels of (p)ppGpp result in low growth rates [30]. However, in this study, we observed an inverse correlation, as the biofilm-forming group of UPECs exhibited a significantly higher basal level of (p)ppGpp and, most remarkably, higher growth rates than the non biofilm-forming strains cultivated under the same non-limiting conditions, which is in stark contrast to the knowledge obtained to date. In a way, our observations are supported by a study on ECOR isolates (an evaluation of the regulatory relationship of (p)ppGpp and RpoS), which showed that regulatory pathways are not uniform within one species [83,84]. Experiments conducted on the K-12 strain suggest that (p)ppGpp should stimulate RpoS synthesis, but results described by Ferenci et al. indicate that RpoS levels are not equally stimulated by high (p)ppGpp in all ECOR isolates [84].

Furthermore, targets that are upregulated during induction of the stringent response may not be sensitive to basal ppGpp levels, which for some reason, are maintained at higher levels in the cell [85,86]. Imholz et al. described that modest increases in (p)ppGpp (<100 pmol OD^−1^) do not appear to immediately inhibit biomass synthesis (except for stable RNA). High concentrations of pseudomonic acid rapidly increased ppGpp accumulation and canonically abruptly stopped growth. On the other hand, low concentrations of pseudomonic acid also triggered ppGpp synthesis (up to 60–100 pmol OD ^−1^) and an immediate, but smaller, decrease in rRNA synthesis. However, the exponential growth rate was not affected in the short term by a moderate increase in ppGpp concentrations [87]. The authors also determined that (p)ppGpp directly inhibits protein synthesis, controlling the rate of ribosome synthesis, albeit only at high concentrations of ppGpp. Potential direct inhibition of translation or ribosome-associated factors is probably not relevant at basal levels of (p)ppGpp [88].

Our results show that the role of (p)ppGpp in bacterial growth and virulence (tested in *G. mellonella* model) may be more complicated. The pleiotropic nature of the mechanism and its association with other regulatory pathways, as well as bacterial physiology and growth conditions, affect this correlation. Further research is needed to understand the relationship between basal (p)ppGpp levels and growth rate.

## 4. Materials and Methods

The strains were isolated and identified in the laboratory of Gdansk Regional Hospital in 2017 and 2021. All bacterial isolates data were anonymized. Of the 118 clinical *E. coli* strains, 93 were from women (78.8%) and 22 from men (18.6%). Patients’ ages ranged from 1 month to 100 years. The reference uropathogenic strains UTI89 and CFT073 were used as controls, making the group size n = 120. Bacteria were grown in Lysogeny Broth (Lennox, Sigma-Aldrich, Darmstadt, Germany) at 37°C, with shaking. For growth rate assays, MOPS minimal medium with low phosphate (0.4 mM) was used. The optical density of cultures was monitored using an EnSpire instrument (Perkin Elmer Singapore Pte. Ltd., Singapore).

### 4.1. Virulence Gene Detection and Phylogenetic Group Determination

The UPEC collection was tested for the presence of virulence factors: FimH, Sfa, PapC, IbeA, HlyA, CNF1, Iss, SitA, the presence of Ag43 antigen, and phylogenetic group membership, which were determined by PCR. All amplified products were visualized in 1% agarose in 0.5%TAE buffer (20 mM Tris pH 8.3, 0.5 mM EDTA, 10 mM acetic acid). RotiSafe dye (CarlRoth) was used to visualize the DNA. The details of primer (Sigma Aldrich) sequences and predicted sizes of amplified products are given in Table 4. UPEC isolates were assigned to separate groups (A, B1, B2, C, D, E, and F), according to Clermont’s classification. The presence or absence of *arpA*/*chuA*/*yjaA*/TspE4.C2 genes was assessed using quadruplex PCR. Separate primers ArpAgpE and trpAgpC were used to determine allele-specific E and C phylogenies, respectively. PCR amplifications were performed in an Eppendorf TM Mastercycler proS thermocycler under the following conditions: initial denaturation at 94 °C for 4 min, 30 cycles of 5 s at 94 °C, and 20 s at 57 °C (E-group), or 59 °C (quadruplex and C-group), 72 °C for 1 min, and final extension at 72 °C for 5 min. Based on the genotypes obtained, the strains were grouped following the principles described by Clermont et al. in 2013 [17]. The presence of virulence factors encoding fimbriae P (*papC*), fimbriae type 1 (*fimH*), fimbriae S (*sfa*), cytotoxic necrotizing factor 1 (*cnf1*), hemolysin (*hlyA*), and aerobactin (*aer*) was determined by PCR, where reaction conditions included initial denaturation for 5 min at 94 °C, 30 cycles of 30 s at 94 °C, 30 s at 60 °C, and 5 min at 72 °C, with a final elongation for 10 min at 72 °C. The reaction conditions for the *iss*, *sitA*, *ibeA*, and *agn*43 primers were as described previously [53,89].

### 4.2. Evaluation of the Biofilm-Forming Phenotype

Biofilm formation of isolates was determined by culturing them on Congo red agar CRA plates, as described previously by Freeman et al. in 1989 [91] (Brain Heart Infusion Agar BHIA medium with 0.08% Congo red, supplemented with 3.6% sucrose). Briefly, bacteria were seeded on plates and then incubated at 37 °C for 24 h. The morphology of bacterial colonies was the basis of the categorization of the strain collection. Biofilm-producing strains formed black colonies, while non-biofilm-forming strains formed red colonies.

### 4.3. Susceptibility Assay

This evaluation was conducted by the disk-diffusion method using commercially available antibiotic disks. It was carried out in accordance with the European Committee for Antimicrobial Susceptibility Testing (EUCAST) guidelines on Mueller–Hilton (MH) agar (Sigma Aldrich, Burlington, MA, USA) [92]. Briefly, a 0.5 McFarland unit (measured by a Densila-Meter II (ErbaLachema, Brno, Czech Republic)) suspension was prepared from overnight culture and spread on MH medium using a sterile swab. The plates were incubated at 37 °C for 24 h, and then zones of growth inhibition were measured. Susceptibility to 16 antibiotics (Biomaxima) was tested: amikacin (AK), nitrofurantoin (F), ampicillin (AM), ceftriaxone (CRO), norfloxacin (NOR), piperacillin (PRL), ciprofloxacin (CIP), gentamicin (CN), cefoxitin (FOX), cefuroxime (CXM), ceftibuten (CFB), meropenem (MEM), fosfomycin (FOS), azithromycin (AZM), trimethoprim/sulfamethoxazole (SXT), and tobramycin (TOB).

### 4.4. Evaluation of (p)ppGpp Accumulation

The intracellular alarmone levels were assessed according to the well-established TLC assay [93]. Briefly, bacterial colonies were suspended in MOPS (4-morpholinopropanesulfonic acid) minimal medium, with a low concentration of phosphate (0.4 mM), to a density of 0.5 by McFarland’s standard, and then cultured for two generations with orthophosphoric acid [P^33^] (0.5 μCi/mL). Samples were harvested over time and lysed with formic acid (13 M) and then frozen twice and centrifuged (5 min, 14000 RPM). Nucleotide extracts were separated by thin-layer chromatography using PEI cellulose plates (Sigma-Aldrich, Darmstadt, Germany) in 1.5 M potassium phosphate buffer (pH 3.4). Chromatograms were analyzed using a Phosphorimager (Typhoon 9200 GE Healthcare, Uppsala, Sweden). QuantityOne (BioRad, Hercules, CA, USA) software was used for densitometric analysis.

### 4.5. The Surrogate Model of Infection

Larvae of the greater moth *Galleria mellonella* (*Lepidoptera: Pyralidae*) were purchased from a livestock supplier (Exoticroom, Łódź, Poland) and were stored in the dark at 20 °C until use. In all experiments, larvae weighing about 250–300 mg, without signs of melanization and devoid of black spots in the epidermis, were used. The insects were infected with bacteria at a rate of 10^4^ to 10^7^/larva using a Hamilton syringe with a blunt-ended needle for the injection in the last left proleg. Control group insects were injected with sterile PBS. The larvae were kept on petri dishes with Wathman blotting paper and incubated at 37 °C in the dark. Observations were made every 24 h for the next three days to detect changes in behavior and melanization. Death was determined by lack of movement and dark pigmentation of the larvae’s epidermis.

### 4.6. Statistics

All experiments were performed in duplicate or triplicate. Statistical analyses were performed with Prism version 8.4.3 (GraphPad Software, San Diego, CA, USA) using Fisher’s two-sided exact test, Pearson’s test, and the Student’s *t*-test. Statistical significance was assumed at *p* < 0.05.

## 5. Conclusions

The results shown in this work present detailed the analysis of our UPEC strain collection. In addition to performing Clermont classification, we analyzed the ability of these strains to form biofilm and discovered a subset in the collection that effectively produced metabolically active biofilms. Most interestingly, isolates from this group exhibited increased intrinsic levels of the stringent response alarmones, (p)ppGpp, which was accompanied by higher growth rates. This finding is in contrast to the knowledge available to date regarding the role of (p)ppGpp in growth rate control. Our data also indicate that (p)ppGpp modulates biofilm formation and virulence in the *G. mellonella* model. Thus, the (p)ppGpp cellular pool is crucial for bacteria and links pathogenesis to their metabolic status.

## Figures and Tables

**Table 1:** UPEC virulence genes categorized according to their occurrence in Clermont’s phylogroups.

Virulence genes	No. (%) of UPEC Isolates Identified in the Clermont Phylogroups								Statistical Analysis of Distribution of Virulence Genes and Phylogenetics		
	A n = 3	B1 n = 13	B2 n = 86	C n = 2	D n = 9	F n = 4	Indefinite n = 3	Total n = 120	B1 ^1^ n = 13	B2 ^1^ n = 86	Fisher’s Exact Test *p* Value (B1 vs. B2)
Adhesins											
*fimH*	3 (100)	13 (100)	86 (100)	2 (100)	9 (100)	4 (100)	3 (100)	120 (100)	ns	ns	ns
*sfa*	2 (67)	6 (46)	66 (77)	1 (50)	5 (56)	2 (50)	2 (66.7)	84 (70)	ns	0.0148	0.0398
*papC*	0	1 (8)	60 (70)	0	5 (56)	2 (50)	0	68 (56.7)	0.0002	<0.0001	<0.0001
Invasin											
*ibeA*	0	1 (8)	22 (26)	0	0	1 (25)	0	24 (20)	ns	0.0207	ns
Toxins											
*hlyA*	0	1 (8)	51 (59)	1 (50)	0	0	0	53 (41.2)	0.0061	<0.0001	0.0006
*cnf*	0	0	29 (34)	0	0	0	0	29 (24.2)	0.0364	<0.0001	0.0093
Iron Acquisition											
*aer*	2 (67)	9 (69)	63 (73)	2 (100)	7 (78)	4 (100)	3 (100)	90 (75)	ns	ns	ns
*sitA*	3 (100)	9 (69)	82 (99)	1 (50)	9 (100)	4 (100)	3 (100)	111(92.5)	ns	ns	0.0097
Serum Resistance											
*iss*	2 (67)	6 (46)	10 (12)	1 (50)	1 (11)	0	2 (67)	22 (18.3)	0.0141	0.0042	0.0061
Biofilm formation											
*agn43a* *agn43b*	0	0	48 (55.8)	0	1 (11.1)	1 (25)	1 (33.3)	51 (42.5)	<0.0001	<0.0001	0.0001
Mean; Range of VF	4 (2–5)	3.52 (2–6)	6.01 (2–10)	4 (3–5)	4.11 (2–6)	4.25 (3–6)	4.66 (4–5)	5.43 (2–10)	<0.0001		

“ns” indicates that the result is not statistically significant. ^1^ The statistical analysis of B1 and B2 individuals was conducted against the whole group of strains (n = 120).

**Table 2:** CRA biofilm formation ability in regard to UPEC phylogenetic groups.

Biofilm Formation by CRA	Phylogenetic Classification by Clermont						
	A n = 3 Count (%)	B1 n = 13 Count (%)	B2 n = 86 Count (%)	C n = 2 Count (%)	D n = 9 Count (%)	F n = 4 Count (%)	Fisher’s Exact Test *p* Value
Biofilm forming (n = 39)	2 (66)	6 (46)	25 (29)	1 (50)	3 (33)	1 (25)	ns
Non-biofilm forming (n = 81)	1 (34)	7 (54)	61 (71)	1 (50)	6 (67)	3 (75)	

**Table 3:** Biofilm-forming ability on CRA vs. the presence of a multiple resistance phenotype.

Group	Number of Resistant Strains (Multiple Resistant)	% of the Group (% of Multiple Resistance within Resistant Strains)	Fisher’s Exact Test *p* Value
Biofilm forming n = 39	16 (12)	30.8% (75%)	0.0410
Non-biofilm forming n = 81	37 (16)	19.7% (43.2%)	

**Table 4:** Primers used in PCR reactions related to phylogenetic clustering and virulence gene detection.

PCR Reaction	ID	Target	Primer Sequence (5′-3′)	Final Concn. (μM)	Product	Reference
Quadruplex	chuA.1b	*chuA*	5′-ATGGTACCGGACGAACCAAC-3′	20	288	[17]
	chuA.2		5′-TGCCGCCAGTACCAAAGACA-3′			
	yjaA.1b	*yjaA*	5′-CAAACGTGAAGTGTCAGGAG-3′		211	
	yjaA.2b		5′-AATGCGTTCCTCAACCTGTG-3′			
	TspE4C2.1b	TspE4.C2	5′-CACTATTCGTAAGGTCATCC-3′		152	
	TspE4C2.2b		5′-AGTTTATCGCTGCGGGTCGC-3′			
	AceK.f	*arpA*	5′-AACGCTATTCGCCAGCTTGC-3′	40	400	
	ArpA1.r		5′-TCTCCCCATACCGTACGCTA-3′			
Group E	ArpAgpE.f	*arpA*	5′-GATTCCATCTTGTCAAAATATGCC-3′	20	301	
	ArpAgpE.r		5′- GAAAAGAAAAAGAATTCCCAAGAG-3′			
Group C	trpAgpC.1	*trpA*	5′-AGTTTTATGCCCAGTGCGAG-3′		219	
	trpAgpC.2		5′-TCTGCGCCGGTCACGCCC-3′			
Internal control	trpBA.f	*trpA*	5′-CGGCGATAAAGACATCTTCAC-3′	12	489	
	trpBA.r		5′-GCAACGCGGCCTGGCGGAAG-3′			
Virulence genes	fimH F	*fimH*	5′ TGCAGAACGGATAAGCCGTGG 3′	1	508	[90]
	fimH R		5′ GCAGTCACCTGCCCTCCGGTA 3′			
	sfa F	*sfa*	5′ CGGAGGAGTAATTACAAACCTGGCA 3′		407	[90]
	sfa R		5′ CTCCGGAGAACTGGGTGCATCTTAC 3′			
	papC F	*papC*	5′ GACGGCTGTACTGCAGGGTGTGGC 3′		328	[90]
	papC R		5′ ATATCCTTTCTGCAGGGATGCAATA 3′			
	hlyA F	*hlyA*	5′ AACAAGGATAAGCACTGTTCTGGCT 3′		1177	[90]
	hlyA R		5′ ACCATATAAGCGGTCATTCCCGTCA 3′			
	aer F	*iucC*	5′-AAACCTGGCTTACCAACTGT-3′		269	[45]
	aer R		5′-ACCCGTCTGCAAATCATGGAT-3′			
	cnf F	*cnf*	5′-TTATATAGTCGTCAAGATGGA-3′		693	[45]
	cnf R		5′-CACTAAGCTTTACAATATTGA-3′			
	iss F	*iss*	5′ GTGGCGAAAACTAGTAAAACAGC 3′		760	[53]
	iss R		5′ CGCCTCGGGGTGGATAA 3′			
	sitA F	*sitA*	5′ AGGGGGCACAACTGATTCTCG 3′		608	[53]
	sitA R		5′ TACCGGGCCGTTTTCTGTGC 3′			
	ibeA F	*ibeA*	5′ AGGCAGGTGTGCGCCGCGTAC 3′		171	[53]
	ibeA R		5′ TGGTGCTCCGGCAAACCATGC 3′			
Multiplex PCR	CFT073a F	*agn43aCFT073*	5′ AGGCAGGAGGAACTGCCAGT 3′	0.25	340	[89]
	CFT073a R		5′ TAAATGAGGGTGTCCCGTGCC 3′			
	CFT073b F	*agn43aCFT073*	5′ CAGCCGGATCTGCGGCACT 3′		440	
	CFT073b R		5′ ACTCTGGTGTTTCTGGCTGTT 3′			

## Data Availability

Not applicable.

## Supplementary Materials

The following supporting information can be downloaded at: https://www.mdpi.com/article/10.3390/ijms24043315/s1.
