# Supplementary material for: Increased Levels of (p)ppGpp Correlate with Virulence and Biofilm Formation, but Not with Growth, in Strains of Uropathogenic Escherichia coli
Source: Int J Mol Sci. 2023 Feb 7;24(4):3315. doi: 10.3390/ijms24043315 (PMC9962837; doi:10.3390/ijms24043315)
Supplement: Supplementary file 1 [file ijms-24-03315-s001.zip › ijms-2144660-supplementary.pdf]

Increased Levels of (p)ppGpp Correlate with Virulence and Biofilm Formation, but Not with Growth, in Strains of Uropathogenic *Escherichia coli*

Supplementary Material

Table S1. Genetic and phenotypic characteristics.

Code	Name	Quantity of virulence factors	AK	F	AM	CRO	NOR	PRL	CIP	CN	FOX	CXM	CFB	MEM	FOS	AZM	SXT	TOB	SENSITIVE	RESISTANE (Quantity)
-	**CFT073**	7																	S	
-	**UTI89**	8																	S	
KB003	M 821	3					+													R (1)
KB004	M 826	6		+	+		+													R (3)
KB005	M 858	3		+	+		+		+											R (4)
KB006	M 911	8			+															R (1)
KB007	M 1079	8																	S	
KB008	M 1132	2																	S	
KB009	M 1146	2																	S	
KB010	M 1627	3																	S	
KB011	M 1648	3																	S	
KB012	M 902	5		+																R (1)
KB013	M 1407	5																	S	
KB014	M 872	7			+															R (1)

KB015	M 1382	3			+		+		+											R (3)
KB016	M 1401	8			+															R (1)
KB017	M 827	7																	S	
KB018	M 819	4		+	+		+		+								+			R (5)
KB019	M 1488	5																	S	
KB020	M 1693	8																	S	
KB021	M 1389	6			+												+			R (2)
KB022	M 946	6																	S	
KB023	M 883	8			+															R (1)
KB024	M 845	9																	S	
KB025	M 948	8			+															R (1)
KB026	M 1684	4			+		+		+								+			R (4)
KB027	M 1428	5			+															R (1)
KB028	M 1619	5			+		+		+								+			R (4)
KB029	M 1636	4																	S	
KB030	M 1437	6																	S	
KB031	M 1611	5																	S	
KB032	M 1107	5		+	+															R (2)
KB033	M 1315	5			+		+		+								+			R (4)
KB034	M 1337	5			+				+											R (2)
KB035	M 1276	4																	S	
KB036	M 1239	5																	S	
KB037	M 1290	8																	S	
KB038	M 1245	9																	S	
KB039	M 1231	5																	S	
KB040	M 1280	5																	S	
KB041	M 2727	4																	S	
KB042	M 2886	4			+												+			R (2)
KB043	M 2716	5																	S	

KB044	M 2846	5																	S	
KB045	M 2804A	5																	S	
KB046	M 2680	2																	S	
KB047	M 2673	3																	S	
KB048	M 2913	3																	S	
KB049	M 2608	4		+	+		+	+									+			R (5)
KB050	M 2688	7			+															R (1)
KB051	M 2626	4			+	+	+		+			+					+			R (6)
KB052	M 2676	3			+												+			R (2)
KB053	M 2691	6																	S	
KB054	M 2653	6																	S	
KB055	M 2581	6			+															R (1)
KB056	M 2639	7																	S	
KB057	M 2578	4																	S	
KB058	M 2590	6																	S	
KB059	M 2763	7																	S	
KB060	M 2765	6																	S	
KB061	M 2851	2		+	+				+	+							+			R (5)
KB062	M 2735	7			+															R (1)
KB063	M 2731	7																	S	
KB064	M 2741	5																	S	
KB065	M 2810	8																	S	
KB066	M 2821	8																	S	
KB067	M 2826	7																	S	
KB068	M 2839	4																	S	
KB069	M 2840	7			+															R (1)
KB070	M 2842	5																	S	
KB071	M 2900	4			+															R (1)
KB072	M 2903	5		+	+															R (2)

KB073	KR 412	6			+	+	+		+			+				+	+	+		R (8)
KB074	KR 492	7	+																	R (1)
KB075	KR 212	6			+	+	+		+			+				+	+	+		R (8)
KB076	KR 662	6			+	+	+		+	+		+				+	+	+		R (9)
KB077	KR 72	5			+	+	+	+	+			+						+		R (7)
KB078	KR 81	5			+															R (1)
KB079	KR 212	5			+															R (1)
KB080	KR 201	5																	S	
KB081	KR 178	4			+		+		+	+							+	+		R (6)
KB082	KR 315	7			+	+	+		+	+		+						+		R (7)
KB083	M 2775	5			+															R (1)
KB084	M 2773	7			+															R (1)
KB085	M 2815	7			+															R (1)
KB086	M 2845	5																	S	
KB087	M 2745	6																	S	
KB088	M 2785	5															+			R (1)
KB089	M 2835	5																	S	
KB090	M 2699	2																	S	
KB091	M 2779	4			+															R (1)
KB092	M 2788	4			+		+		+								+			R (4)
KB093	M 2717	6			+															R (1)
KB094	M 2804B	3			+												+			R (2)
KB095	M 2759	5																	S	
KB096	M 2812	7																	S	
KB097	M 2860	6																	S	
KB098	M 3010	5																	S	
KB099	M 2087	5																	S	
KB100	M 2098	4			+															R (1)
KB101	M 2057	7																	S	

KB102	M 2060	4			+	+	+		+			+					+	+		R (7)
KB103	M 1788	6																	S	
KB104	M 2045	5			+		+		+											R (3)
KB105	M 2227	6																	S	
KB106	M 2235	8			+															R (1)
KB107	M 2194	5			+															R (1)
KB108	M 2224	6																	S	
KB109	M 2187	5																	S	
KB110	M 2193	3			+		+		+								+			R (4)
KB111	M 2178	6																	S	
KB112	M 2182	7																	S	
KB113	M 2168	5																	S	
KB114	M 2170	6																	S	
KB115	M 2144	8																	S	
KB116	M 2162	5																	S	
KB117	M 2128	6																	S	
KB118	M 2131	10																	S	
KB119	M 2107	3																	S	
KB120	M 2112	7			+												+			R (2)
																		**Total**	**67 (55.8%)**	**53 (44.2%)**

The following antimicrobial agents were used: amikacin (AK), nitrofurantoin (F), ampicillin (AM), ceftriaxone (CRO), norfloxacin (NOR), piperacillin (PRL), ciprofloxacin (CIP), gentamicin (CN), cefoxitin (FOX), cefuroxime (CXM), ceftibu-ten (CFB), meropenem (MEM), fosfomycin (FOS), azithromycin (AZM), trimethoprim/sulfmetaxazole (SXT), and tobramycin (TOB). The non-biofilm-forming strains were marked in black and biolfilm forming ones in red.

Table S2. Table of co-occurrence of virulence factors. Statistically significant results ($p < 0.05$) from Fisher's exact test and the direction of dependence determined by Pearson's test (Pearson's correlation coefficient r is shown in parentheses) are shown in green.

	papC	*hlyA*	*aer*	*cnf*	*iss*	*sitA*	*ibeA*	*agn43*
sfa	0.0002 (0.345)	<0.0001 (0.3625)	ns	0.0001 (0.3271)	0.0002 (−0.2068)	ns	ns	0.0437 (0.1950)
papC		<0.0001 (0.5407)	ns	<0.0001 (0.3758)	0.036 (−0.2810)	ns	ns	0.002 (0.3776)
hlyA			ns	<0.0001 (0.6347)	<0.0001 (-0.3780)	ns	ns	<0.0001 (0.3895)
aer				0.0788 (−0.1915)	ns	0.0365 (0.0183)	ns	ns
cnf					0.0245 (−0.2172)	ns	ns	0.00051 (0.2628)
iss						ns	0.0348 (0.0339)	0.0001 (−0.3638)
sitA							ns	ns
ibeA								ns

ns – non significant
